# Highly Multiplexed Proteomic Analysis of Quantiferon Supernatants To Identify Biomarkers of Latent Tuberculosis Infection

**DOI:** 10.1128/JCM.01646-16

**Published:** 2017-01-25

**Authors:** Mary Ann De Groote, Michael Higgins, Thomas Hraha, Kirsten Wall, Michael L. Wilson, David G. Sterling, Nebojsa Janjic, Randall Reves, Urs A. Ochsner, Robert Belknap

**Affiliations:** a SomaLogic, Inc., Boulder, Colorado, USA; b Mycobacteria Research Laboratories, Department of Microbiology, Immunology and Pathology, Colorado State University, Fort Collins, Colorado, USA; c Department of Pathology and Laboratory Services, Denver Health, Denver, Colorado, USA; d Denver Health and Hospital Authority and Denver Public Health, Denver, Colorado, USA; Carter BloodCare and Baylor University Medical Center

**Keywords:** biomarkers, diagnosis, immunity, latent infection, proteomics, tuberculosis

## Abstract

The tests for diagnosing latent tuberculosis infection (LTBI) are limited by a poor predictive value for identifying people at the highest risk for progressing to active tuberculosis (TB) and have various sensitivities and specificities in different populations. Identifying a more robust signature for LTBI is important for TB prevention and elimination. A pilot study was conducted with samples from immigrants to the United States that were screened for LTBI by the three commercially approved tests, namely, the tuberculin skin test (TST), the Quantiferon-TB Gold in-tube (QFT-GIT), and the T-SPOT.TB (T-SPOT). QFT-GIT supernatants from 13 people with concordant positive results and 26 people with concordant negative results were analyzed via the highly multiplexed SOMAscan proteomic assay. The proteins in the stimulated supernatants that distinguished LTBI from controls included interleukin-2 (IL-2), monocyte chemotactic protein 2 (MCP-2), interferon gamma inducible protein-10 (IP-10), interferon gamma (IFN-γ), tumor necrosis factor superfamily member 14 (TNFSF14, also known as LIGHT), monokine induced by gamma interferon (MIG), and granzyme B (*P* <0.00001). In addition, antigen stimulation increased the expression of heparin-binding EGF-like growth factor (HB-EGF) and activin AB in LTBI samples. In nil tubes, LIGHT was the most significant marker (*P* <0.0001) and was elevated in LTBI subjects. Other prominent markers in nonstimulated QFT-GIT supernatants were the complement-3 components C3b, iC3b, and C3d, which were upregulated in LTBI and markedly decreased upon stimulation. We found known and novel proteins that warrant further studies for developing improved tests for LTBI, for predicting progression to active disease, and for discriminating LTBI from active TB.

## INTRODUCTION

Tuberculosis (TB) is a major global health problem, with an estimated 2 billion people infected with Mycobacterium tuberculosis worldwide. From this large reservoir, millions of people develop TB disease, and 10.4 million TB incident cases were reported in 2015 (1). Proper and accurate identification and treatment of latent TB infection (LTBI) can reduce substantially the risk of developing TB and is a major focus of TB control in the United States and in TB programs around the world (1, 2).

There is no gold standard available for diagnosing LTBI. Hence, there is no way to firmly determine the sensitivity and specificity of tests designed to detect TB infection. Three tests are currently commercially available to diagnose TB infection, including two interferon gamma (IFN-γ) release assays (IGRAs; T-SPOT and QFT-GIT) and the tuberculin skin test (TST) (2). The TST measures cell-mediated immunity in the form of a delayed-type hypersensitivity response to the most commonly used purified protein derivative (PPD) of Mycobacterium tuberculosis. Until the commercial IGRAs were available, the TST was the only test for diagnosing LTBI but with several well-described limitations, for example, the need for two visits, the subjective quality of the results, the low sensitivity for active TB, and the occurrence of false-positive results due to prior BCG vaccination or nontuberculous mycobacteria (NTM) infection (3–5). The IGRAs were developed to overcome many of these limitations. They require a single blood draw and assess the cell-mediated immune response by measuring IFN-γ produced after an incubation with TB-specific antigens, but these tests also have their limitations, including a low predictive value for identifying patients with the greatest immediate risk for developing active TB (6–8). Furthermore, IGRAs have high rates of false-positive results in some lower-risk, non-BCG vaccinated populations, and various sources of variability can impact the reproducibility (3, 4, 9). The need for better TB diagnostic tests is recognized as an important component for improving global TB control and prevention (10, 11).

We sought to identify proteins that highly correlate with LTBI to improve tests for diagnosing LTBI. This pilot study applied a highly multiplexed proteomic discovery platform (SOMAscan), which relies on modified DNA aptamers selected with high affinity to >4,000 protein targets to uncover promising protein markers (12). Since the multiplexed platform has not been validated using matrices such as supernatants from extended stimulation tubes and controls, we studied how reliable such a matrix is for biomarker discovery. Toward these aims, we utilized residual QFT-GIT samples from the TB Epidemiologic Studies Consortium (TBESC) parent study in a feasibility study to determine the performance of the SOMAscan assay with heparin plasma from both the nil and M. tuberculosis antigen (Mtb)-stimulated tubes.

## RESULTS

### Study subjects and sample quality assessment.

The sample groups of 13 triple-positive and 26 triple-negative subjects were well balanced with respect to sex and ethnicity (Table 1). The differences in the total protein abundances between the four sample groups (LTBI versus healthy control [HC] and stimulated versus nil) were not significant in any of the three dilutions employed in the assay, as reflected in the narrow distribution of scale factors for median normalization prior to data analysis (13) (see Fig. S1 in the supplemental material). The SOMAscan run included one buffer control, four quality controls, and five pooled calibrator plasma samples. Calibrators were pooled samples consistent with the matrix of the clinical samples, which were used to correct for plate-to-plate variations.

**TABLE 1:** Characteristics of participants from whom QFT-GIT supernatants were used for SOMAscan analysis in this study

Subject characteristics	LTBI (*n* = 13)	HC (*n* = 26)
Male (%)	53.8	50
Age (years [median, range])	32 (26–35)	29 (18–59)
Black/African American (%)	46.2	50
Asian (%)	46.2	38.5

### SOMAscan and ELISA results for IFN-γ.

**FIG 1:**
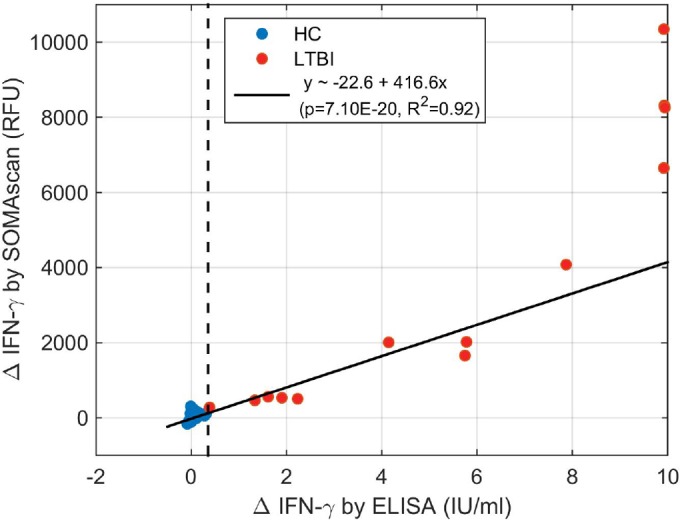
Correlation of Qiagen QFT-GIT ELISA with SOMAscan data for differential IFN-γ signals between antigen and nil tubes. QFT-GIT supernatants in LTBI are in red and supernatants from HC are in blue. All samples from HC had <0.35 IU/ml and <400 RFU of IFN-γ released upon stimulation. Measurements of IFN-γ release in LTBI samples correlated well between ELISA and SOMAscan data, though ELISA has a smaller dynamic range with an upper limit at 10 IU/ml.

IFN-γ is one of the >4,000 analytes measured by the SOMAscan assay. In the nil tube supernatants of the study subjects, the median IFN-γ signal was 1,434 relative fluorescence units (RFU), and no significant differences were noted between LTBI (1,494 RFU) and HC (1,414 RFU). For reference, the mean (± standard deviation) RFU value of control sequences with compositions similar to modified DNA aptamers but with no known binding affinity to any of the proteins was 328 ± 53 RFU. In the supernatants of the stimulated tubes, the median IFN-γ signal was elevated for LTBI subjects (3,589 RFU) compared with that of HC subjects (1,419 RFU). The IFN-γ SOMAscan data correlated well with the measurements of IFN-γ via the commercial enzyme-linked immunosorbent assay (ELISA) over a concentration range of 0 to 8 IU/ml (Fig. 1). The commercial ELISA has 10 IU/ml as the upper limit of titration, with 1 IU representing 40 pg/ml (unpublished communication, Qiagen). For the QFT-GIT assay, a difference of 0.35 IU/ml between the IFN-γ levels measured in the stimulated and nil tube supernatants is the cutoff for diagnosing TB infection.

### Markers in stimulated plasma.

**FIG 2:**
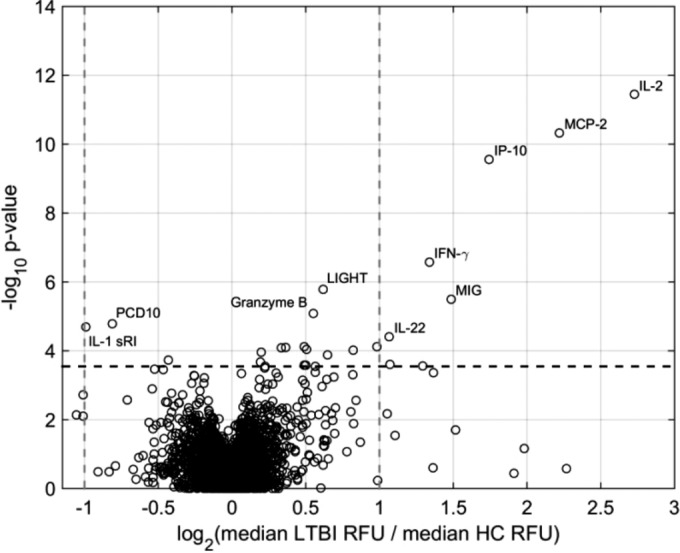
Volcano plot of the differential expression of proteins measured in stimulated QFT-GIT supernatants in LTBI compared with those in HC. The most statistically significant markers are shown toward the top, and proteins with the largest median up- or downregulation are toward the right and left, respectively.

**FIG 3:**
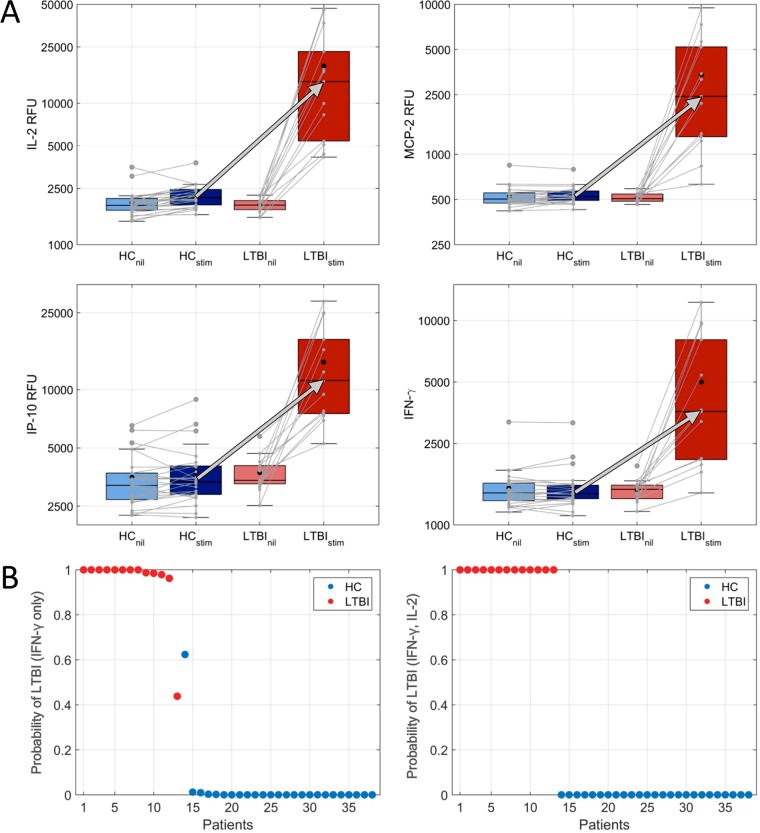
(A) Top markers distinguishing LTBI and HC in stimulated QFT-GIT supernatants. Box plots show IL-2, MCP-2, IP-10, and IFN-γ levels in the supernatants from nil and stimulated tubes in LTBI and HC, along with the median upregulation of these proteins in LTBI (arrows). The association of the paired nil and stimulated samples from the 26 HC and 13 LTBI subjects is indicated by the gray connectors. (B) Models calculating the probability of LTBI were fit using the log-transformed differences between the nil and stimulated tubes. Using only IFN-γ RFU data, there is no decision boundary that perfectly separates the LTBI from HC (left). However, simply adding IL-2 to the model creates perfect separation (right).

One of our Mtb pathogen-specific SOMAmers robustly detected a peptide of the ESAT-6-like protein EsxB (also known as CFP10) in all supernatants from the stimulated tubes only (see Fig. S2). EsxB is one of the antigens used to coat the QFT-GIT tubes, and its consistent, partial release during incubation in the IGRA provided an internal control for sample identity and validated this SOMAmer target. The comparison of the signal levels for the 4,316 analytes measured by SOMAscan in the stimulated supernatants revealed 431 proteins that were significantly different between LTBI and HC at a 5% false-discovery rate (FDR; *q*) (257 increased and 174 decreased with LTBI) and 135 proteins that were significantly different at a 1% FDR (86 increased and 49 decreased with LTBI). The significance and the median fold change of the differential protein expressions between LTBI and HC are depicted in a volcano plot (Fig. 2), and the 16 proteins with the most significant differential expression (*P* <10^−4^) are listed in Table 2. Remarkably, three proteins, namely interleukin-2 (IL-2), monocyte chemotactic protein 2 (MCP-2), and interferon gamma inducible protein-10 (IP-10), were considerably stronger markers of LTBI than IFN-γ, and their expression increased roughly an order of magnitude upon stimulation (Fig. 3A). When calculating the probability of LTBI using proteomic measurements, adding IL-2 to IFN-γ perfectly separated the LTBI subjects from the HC subjects (Fig. 3B). IL-2, MCP-2, and IP-10 were significantly correlated to IFN-γ (ρ >0.8), whereas other strong markers showed much less correlation with IFN-γ, such as granzyme B, protocadherin 10 (PCD10), interleukin 1 sR1, and complement C3b (C3b) (Table 2).

**TABLE 2:** Top markers, ranked by statistical significance, in stimulated QFT-GIT supernatants of LTBI versus HC

Protein Marker	UniProt no.	*t* stat^*a*^	*P* value	*q* value	KS distance^*a*^	IFN-γ correlation (ρ)
IL-2	P60568	10.09	3.60 × 10^−12^	1.55 × 10^−8^	1.00	0.88
MCP-2	P80075	9.17	4.66 × 10^−11^	1.01 × 10^−7^	0.96	0.90
IP-10	P02778	8.55	2.74 × 10^−10^	3.94 × 10^−7^	0.88	0.80
IFN-γ	P01579	6.28	2.66 × 10^−7^	2.87 × 10^−4^	0.81	1.00
LIGHT	O43557	5.69	1.65 × 10^−6^	1.42 × 10^−3^	0.69	0.50
MIG	Q07325	5.48	3.16 × 10^−6^	2.27 × 10^−3^	0.81	0.74
Granzyme B	P10144	5.18	8.17 × 10^−6^	5.03 × 10^−3^	0.69	0.24
PCD10	Q9P2E7	−4.95	1.65 × 10^−5^	8.88 × 10^−3^	−0.65	−0.02
IL-1 sRI	P14778	−4.89	2.00 × 10^−5^	9.61 × 10^−3^	−0.62	−0.34
IL-22	Q9GZX6	4.67	3.89 × 10^−5^	1.68 × 10^−2^	0.65	0.73
C3b	P01024	4.45	7.51 × 10^−5^	2.54 × 10^−2^	0.69	0.26
IL-17	Q16552	4.45	7.63 × 10^−5^	2.54 × 10^−2^	0.65	0.58
Holo-TC I	P20061	4.44	7.95 × 10^−5^	2.54 × 10^−2^	0.62	0.35
iC3b	P01024	4.42	8.25 × 10^−5^	2.54 × 10^−2^	0.58	−0.05
C3d	P01024	4.39	9.06 × 10^−5^	2.56 × 10^−2^	0.69	−0.06
CHRD	Q9H2X0	4.38	9.47 × 10^−5^	2.56 × 10^−2^	0.65	0.29

a Negative values indicate decreased expression in LTBI compared with than in HC. IL, interleukin; MCP-2, monocyte chemotactic protein 2; IP-10, interferon gamma inducible protein-10; IFN-γ, interferon gamma; LIGHT, also known as tumor necrosis factor superfamily member 14 (TNFSF14); MIG, monokine induced by gamma interferon and granzyme B; PCD10, protocadherin 10; Holo-TC I, transcobalamin I; CHRD, chordin; iC3b, complement C3b, inactivated.

### Markers in unstimulated plasma.

**FIG 4:**
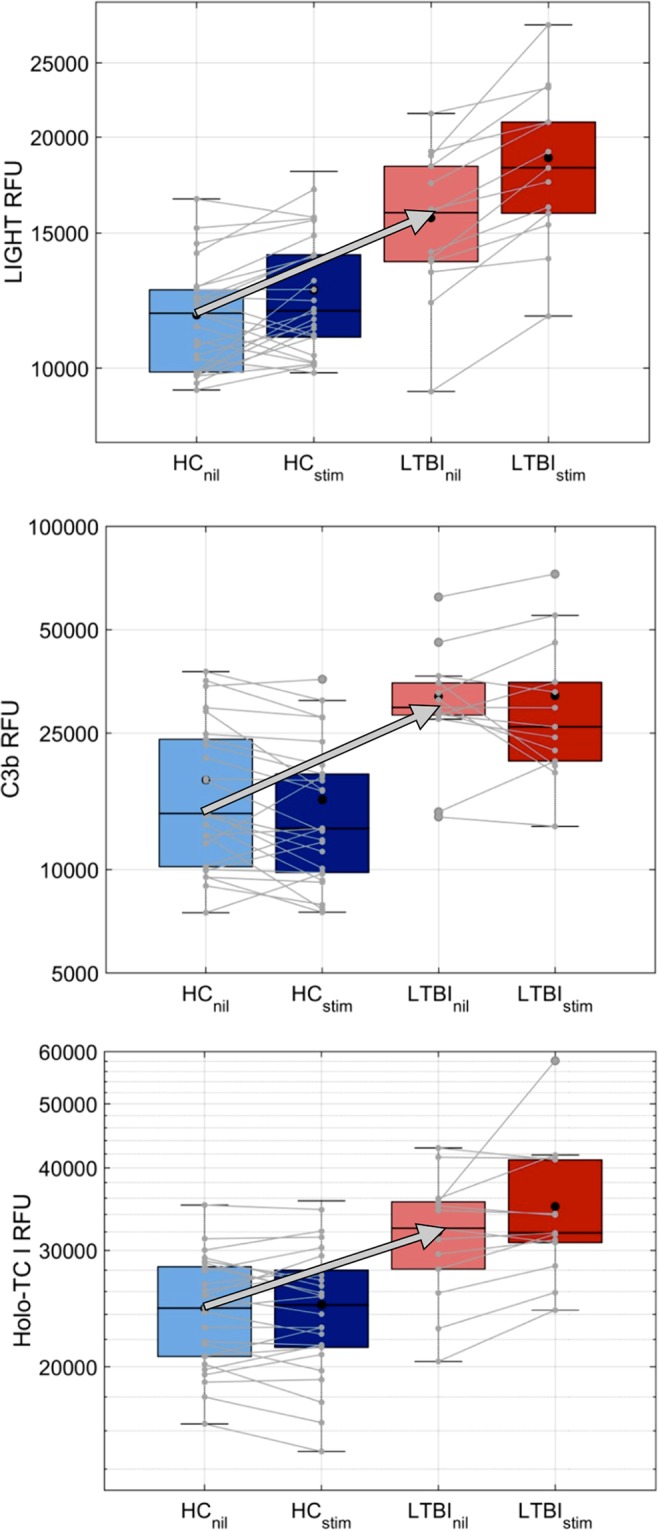
Top markers distinguishing LTBI and HC in nonstimulated (nil) QFT-GIT supernatants. Box plots of LIGHT, C3b, and Holo-TC I levels in QFT-GIT supernatants from nil and stimulated tubes in LTBI and HC, along with the median upregulation of these proteins in LTBI (arrows). The association of the paired nil and stimulated samples from the 26 HC and 13 LTBI subjects is indicated by the gray connectors.

In unstimulated plasma (nil tube), 11 proteins were elevated in LTBI subjects compared to those in HC with *P* values of <0.001, including LIGHT, C3b, inactivated C3b (iC3b), transcobalamin I (Holo-TC I), NHL repeat containing 3 (NHLC3), calnexin, double-stranded RNA-binding nuclear protein 76 (DRBP76), l-plastin, complement 3d, C1-esterase inhibitor, and ubiquitin conjugating enzyme G2 (UB2G2) (Table 3). While the FDR was nearly 40% for most of these markers, LIGHT was the most significant marker that distinguished LTBI from HC in unstimulated supernatants (*P* = 0.000065, *q* = 0.28) and was the fifth top-ranking marker in the above analysis of stimulated samples. The level of LIGHT expression was higher in LTBI than in HC samples and further increased upon stimulation in the LTBI group (Fig. 4). C3b and Holo-TC I were expressed at higher levels in LTBI than in HC regardless of whether the samples were stimulated or not (Fig. 4).

**TABLE 3:** Top markers, ranked by statistical significance, that increased in unstimulated (nil tube) supernatants of LTBI versus HC

Protein Marker	UniProt no.	*t* stat	*P* value	*q* value	KS distance
LIGHT	O43557	4.52	6.5 × 10^−5^	0.28	0.69
C3b	P01024	3.89	4.13 × 10^−4^	0.37	0.65
iC3b	P01024	3.88	4.24 × 10^−4^	0.37	0.72
Holo-TC I	P20061	3.79	5.61 × 10^−4^	0.37	0.57
NHLC3	Q5JS37	3.75	6.20 × 10^−4^	0.37	0.53
Calnexin	P27824	3.70	7.10 × 10^−4^	0.37	0.53
DRBP76	Q12906	3.69	7.46 × 10^−4^	0.37	0.61
l-plastin	P13796	3.68	7.54 × 10^−4^	0.37	0,57
C3d	P01024	3.67	7.75 × 10^−4^	0.37	0.61
C1-esterase inhibitor	P05155	3.62	9.04 × 10^−4^	0.37	0.57
UB2G2	P60604	3.60	9.47 × 10^−4^	0.37	0.48

### Markers in the stimulated versus unstimulated plasma.

**FIG 5:**
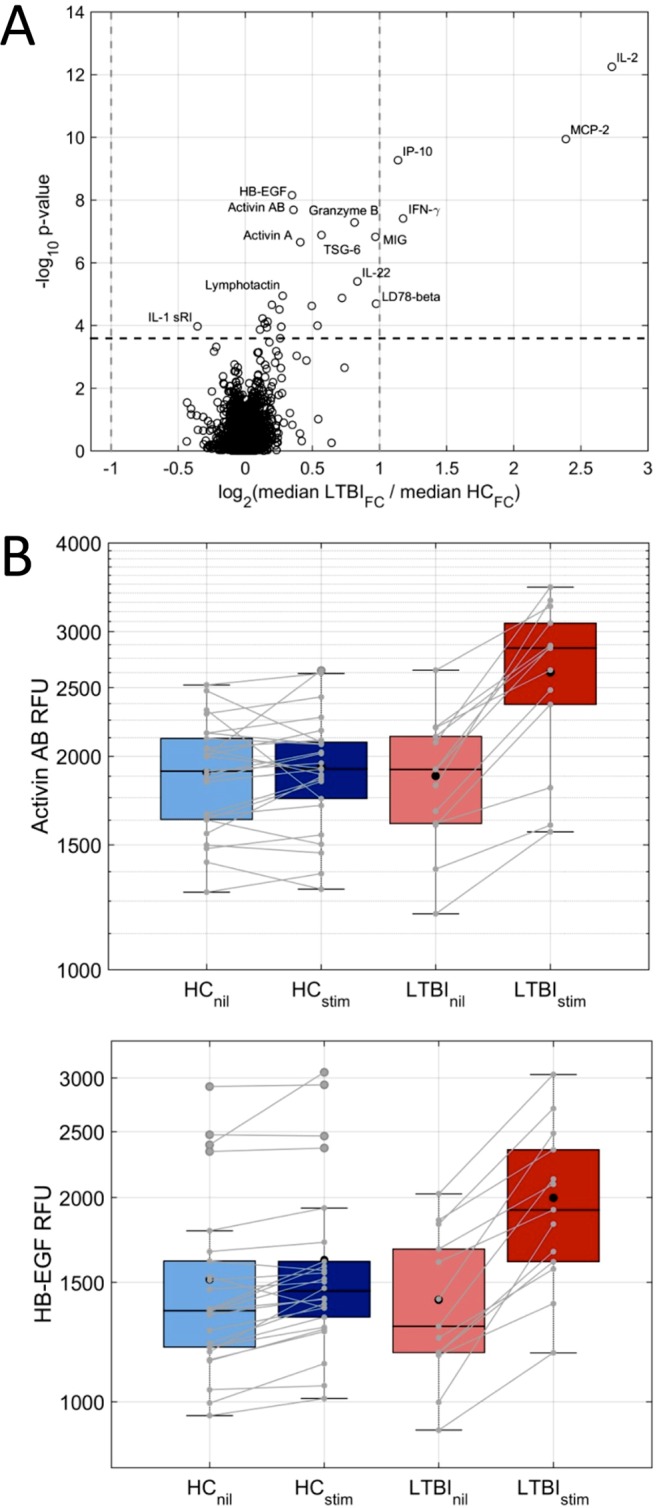
(A) Volcano plot of markers that distinguish LTBI from HC based on the fold change (FC) of differential expression in stimulated versus unstimulated samples. The most statistically significant markers are shown toward the top, and proteins with the largest median ratios of up- or downregulation in LTBI compared with those in HC are toward the right and left, respectively. (B) Box plots of two additional markers, activin AB and HB-EGF, in QFT-GIT supernatants from nil and stimulated tubes in LTBI and HC (IL-2, MCP-2, IP-10, and IFN-γ box plots are shown in Fig. 3).

Lastly, we analyzed the proteomic measurements in the LTBI versus HC samples with regard to the extent of up- or downregulation upon stimulation. This comparison of the ratios (fold change) of the measurements in stimulated to those in unstimulated blood confirmed the three top markers (IL-2, MCP-2, and IP-10) that distinguished LTBI from HC in the direct comparison of stimulated samples, and revealed two additional markers (heparin-binding EGF-like growth factor [HB-EGF] and activin AB) that were altered more significantly than IFN-γ (Fig. 5). The top 20 markers that showed the largest differential responses to stimulation between LTBI and HC are listed in Table 4.

**TABLE 4:** Top 20 markers distinguishing LTBI from HC based on comparing protein expression ratios between stimulated and unstimulated samples

Protein marker	UniProt no.	*t* stat	*P* value	FDR
IL-2	P60568	10.922	5.59 × 10^−13^	2.41 × 10^−9^
MCP-2	P80075	8.944	1.12 × 10^−10^	2.42 × 10^−7^
IP-10	P02778	8.392	5.38 × 10^−10^	7.74 × 10^−7^
HB-EGF	Q99075	7.52	6.91 × 10^−9^	7.46 × 10^−6^
Activin AB	P08476, P09529	7.157	2.04 × 10^−8^	1.76 × 10^−5^
IFN-γ	P01579	6.947	3.86 × 10^−8^	2.78 × 10^−5^
Granzyme B	P10144	6.848	5.20 × 10^−8^	3.21 × 10^−5^
TSG-6	P98066	6.548	1.30 × 10^−7^	7.00 × 10^−5^
MIG	Q07325	6.502	1.49 × 10^−7^	7.15 × 10^−5^
Activin A	P08476	6.378	2.18 × 10^−7^	9.42 × 10^−5^
IL-22	Q9GZX6	5.436	3.94 × 10^−6^	1.55 × 10^−3^
Lymphotactin	P47992	5.091	1.14 × 10^−5^	4.09 × 10^−3^
MIP-1α	P10147	5.04	1.33 × 10^−5^	4.42 × 10^−3^
LD78-β	P16619	4.906	2.00 × 10^−5^	6.16 × 10^−3^
NKG2E	Q07444	4.877	2.18 × 10^−5^	6.28 × 10^−3^
MCP-1	P13500	4.849	2.38 × 10^−5^	6.43 × 10^−3^
Granzyme B^*a*^	P10144	4.769	3.04 × 10^−5^	7.71 × 10^−3^
Lymphotoxin α1/β2	P01374, Q06643	4.55	5.89 × 10^−5^	1.41 × 10^−2^
Granzyme A	P12544	4.47	7.49 × 10^−5^	1.70 × 10^−2^
CST8	O60676	4.42	8.71 × 10^−5^	1.88 × 10^−2^

a Second data set as two separate SOMAmer reagents for granzyme B were included. Activin AB, inhibin beta A chain:inhibin beta B chain heterodimer; TSG-6, TNF-alpha induced protein 6; MIP-1α, macrophage inflammatory protein 1-alpha; LD78, chemokine (C-C motif) ligand 3-like 3; NKG2E, killer cell lectin like receptor C3; CST8, cystatin 8.

We did not detect Mtb pathogen proteins, such as EsxB, in the nil plasma, so it is unlikely that there is a high concentration of circulating pathogen proteins in LTBI. However, we strongly detected the Mtb used in the test, proving this SOMAmer is specific for the target. We did not detect signal with the ESAT-6 SOMAmer, likely because the material in the antigen-stimulated tubes is peptide fragments that do not contain the conformational epitopes used to generate this SOMAmer. We did not include a SOMAmer directed at the Mtb 7.7 peptide on the menu used in this study.

## DISCUSSION

No gold standard test exists for LTBI. The T-SPOT and QFT-GIT were originally approved using active TB to estimate the sensitivity and using individuals at low risk of TB exposure to estimate the specificity. Given the lack of a true standard, we used the best surrogate by including individuals who were either triple negative or triple positive by the three commercially available tests. Our study is unique given the selection of individuals with “confirmed LTBI” with triple-positive test results and documented “no LTBI” with triple-negative results in the same population (e.g., not comparing foreign-born cases and healthy U.S.-born students). Others have performed multiplex analyses of cytokines, but to our knowledge, this is the largest analyte study using supernatants from QFT-GIT (14).

In this pilot study, we detected a number of protein changes in stimulated plasma from QFT-GIT supernatants, including cytokines and chemokines (IL-2, Il-1Ra, IL-10, and IP-10) that have been detected previously (15–22). We were not able to confirm all proteins in LTBI that have been identified (18, 20). We confirmed that IFN-γ is a robust marker of LTBI (which, in a sense served as a positive control), and at the same time we discovered several additional interesting and more promising markers. Some top markers were not correlated with IFN-γ, suggesting targets outside the IFN-γ pathways that might be amenable to further study.

LIGHT was a strong marker of LTBI discovered in both stimulated and unstimulated plasma. This homolog to lymphotoxin has been shown to provide a role in early response to M. tuberculosis infection (23, 24). The fact that it is differentially expressed with and without stimulation suggests that it might be a marker of LTBI or perhaps indicates an innate susceptibility to LTBI (Fig. 4). In addition, a marker that is not dependent on antigen stimulation may serve as an intra-assay control to minimize the possibility of false-negative results in the event of inadequate stimulation.

The overall pattern of protein expression in the supernatants is consistent with immune system activation. In the stimulated tubes, we saw significant enrichment for expected cytokines (IFN-γ and IL-2) and chemokines (IP-10 and MCP-2), likely originating in activated monocytes and T cells. In the nonstimulated tubes, the complement system predominated. Complement component 3 (C3) is a central component of both the classical and alternative activation pathways, and receptors are found on peripheral blood mononuclear cells. C3bi and C3b have been shown to directly bind Mtb, are important molecules in innate recognition of the pathogens (25), and appear as significant markers in both the stimulated and nonstimulated supernatants in LTBI. In HC samples, the levels of C3b decreased with stimulation. LIGHT, or tumor necrosis factor (TNF) superfamily member 14 (TNFSF14), discovered in both stimulated and nonstimulated plasma samples, is a cytokine that binds to TNF receptor SF3 (TNFRSF3)/lymphotoxin beta receptor (LTBR), and binding to the decoy receptor TNFRSF6B modulates its effects. LIGHT activates NF-κB and stimulates the proliferation of T cells. The finding of a higher abundance of this TNF superfamily in the nil tube suggests the presence of markers for predicting risk of LTBI (and perhaps active disease), as has been proposed by others (14).

There are limitations to this study. First, this study included a small number of subjects from a limited number of geographical areas, and no children or HIV-infected individuals were enrolled. A second limitation is that the assay conditions for the SOMAscan assay were developed primarily for serum or plasma samples. Stimulated supernatant is not a matrix that has been studied before, hence, the dynamic ranges of linearity for each SOMAmer were not optimized. Despite this, we identified several of the proteins detected by others and many other potential alternative markers to IFN-γ. In addition, we robustly detected EsxB in the tube itself.

The aim of this pilot study was to determine if there are better protein signatures to distinguish individuals with LTBI from those who are not infected. These findings could potentially lead to developing more accurate diagnostic tests that include combinations of biomarkers. Such a platform could be used in future studies to identify individuals who are at the greatest risk of progressing to active TB disease (26). However, given that it is not ethical to withhold preventative therapy, this type of study will be challenging unless stored serial samples of QFT-GIT supernatants are available from high-priority prospective studies, such as that recently published by Zak and colleagues (27). Additionally, we would like to apply these signatures to subjects with discordant test results (positive for one or two tests [T-SPOT, QFT-GIT, or TST]) (28, 29) to determine if the use of multiple biomarkers can resolve such discrepancies.

The SOMAscan assay is complex and requires sophisticated instrumentation that is not practical for routine use in a patient-near context. However, the knowledge gained from SOMAscan can help pave the way for a simpler platform that might improve on the efficiency, labor-intensiveness, and cost-effectiveness of a test for LTBI. These improvements would go a long way toward TB prevention and elimination efforts. For instance, an IP-10 ELISA performed well on dried plasma spots (30) showing that samples that do not require special handling might be adequate for field testing. An assay simpler than ELISA, with SOMAmers substituted for antibodies (because of their superior heat stability), could be developed that would hopefully lower the cost of the assay and increase the availability of such tests across the globe without a cold-chain requirement.

A major limitation of the current tests (TST and IGRAs) is their inability to predict the development of active TB disease in the future. A positive TST or IGRA result could indicate only a lasting immune response and not necessarily infection with viable bacilli. Evaluating different markers or protein expression profiles like those identified in this study could lead to better tests or algorithms using specific patterns of protein changes that distinguish LTBI from active TB disease and predict progression. The results of this study need to be confirmed and expanded upon in other studies. If the results are confirmed and found to be reproducible, further studies will be needed that include subjects from different geographic regions, children, patients with immune system suppression (especially patients with HIV infection), and pregnant women.

## MATERIALS AND METHODS

### Study design.

The study used QFT-GIT supernatants that had been stored from participants enrolled in a parent study of the TB Epidemiologic Studies Consortium (TBESC) (https://clinicaltrials.gov/ct2/show/NCT01622140), a multicenter research consortium funded by the Centers for Disease Control and Prevention (CDC). The main study was designed to compare the performance of the TST and both licensed IGRAs in patients at increased risk for LTBI. The study “Prospective comparison of the tuberculin skin test and interferon-gamma release assays in diagnosing infection with Mycobacterium tuberculosis and in predicting progression to tuberculosis” was approved by the CDC institutional review board (IRB) as CDC protocol 6293.0, and approved by the Colorado multiple IRB (COMIRB) 12-0802 by deferral to the CDC IRB. The approval for using the residual samples for this study was obtained from the TBESC parent study investigators.

We enrolled individuals living in the United States who were at increased risk of TB and were already being tested for TB infection. Blood was drawn and processed according to the manufacturers' directions for QFT-GIT and T-SPOT. A TST was placed after the blood draw and read 2 to 3 days later. The test results and other data were entered into a secure password-protected database under a study number without names, and a file linking the names with the study number was kept in a locked file cabinet. All data were deidentified and samples were labeled with an alphanumeric code before they were shipped to SomaLogic.

### Sample collection and test procedures.

Select samples from QFT-GIT were frozen immediately after the test was run for potential future evaluations or quality control testing. From samples collected and stored from the parent study, we used residual QFT-GIT samples from two specific types of participants. We included 13 (7 male and 6 female) triple-positive subjects and 26 (13 male and 13 female) triple-negative subjects that presented for immigration screening and were found to have no evidence of active TB after clinical evaluation and chest radiography. The participants originated from 13 different countries in Africa, Asia, and in the Middle East. The sample groups were well balanced with respect to sex and ethnicity. In this study, the QFT-GIT blood test was performed prior to the TST to avoid any booster effect. The QFT-GIT test was processed according to the manufacturer's specifications and the current clinical practice. Each mitogen tube contained phytohemagglutinin and each TB antigen tube contained peptides from three Mtb (ESAT-6, CFP-10, and TB 7.7), whereas the nil tube contained no antigens or mitogens. While data from each of the three tubes was used to interpret the test, only the nil tube and TB antigen tube supernatants were run on SOMAscan. Immediately after incubating at 37°C (see below), 100 μl aliquots were frozen at −70°C in cryopreservation tubes. Separate aliquots of the nil (unstimulated) and Mtb-stimulated QFT-GIT tubes were frozen after complete processing. For this pilot study, Denver Public Health investigators identified residual samples from 13 subjects who tested positive by all 3 tests (TST, QFT-GIT, and T-SPOT) and were therefore likely to have true LTBI and from 26 subjects who tested negative by all 3 tests and were considered free of TB infection, which are referred to as healthy controls (HC).

The SOMAscan assay measures >4,000 proteins simultaneously in a small volume (50 μl) of plasma or serum, has an overall dynamic range of ∼8 logs, a median lower limit of detection of 40 fM, and high precision (5% coefficient of variation [CV]). Plasma was used for this study, and plasma dilutions (0.005%, 1%, and 40%) were applied to capture low-, medium-, and high-abundant proteins. The nomenclature herein refers to the target used to select the reagent from modified nucleotide libraries, which introduce functional groups, typically hydrophobic moieties, that are often found in protein-protein interactions, antibody-antigen interactions, and interactions between small-molecule drugs with their protein targets, but are absent in natural nucleic acids (31). SOMAmer reagents were selected for roughly 4,000 human proteins (47% secreted proteins, 28% extracellular domains, 25% intracellular proteins) that belong to broad biological groups, including receptors, kinases, cytokines, proteases, growth factors, protease inhibitors, hormones, and structural proteins. Median normalization was used to adjust for sample-specific assay bias. Quality control (QC) and calibrators samples were EDTA plasma, in contrast to QFT-GIT supernatants, which were heparin plasma.

### Statistical analysis.

All data were log-transformed to stabilize the variance. Nonparametric statistical tests were used for all comparisons, including the Kolmogorov-Smirnov (KS) test for cross-sectional comparisons and the Kruskal-Wallis test for inter-tube/dilution comparisons within each diagnostic category. Student's *t* tests were used to identify differentially expressed SOMAmer reagents. Benjamini and Hochberg false-discovery rates (FDR) were used to adjust *P* values for multiple comparisons.

## Supplementary Material

Supplemental material
