# Supplementary material for: Highly Multiplexed Proteomic Analysis of Quantiferon Supernatants To Identify Biomarkers of Latent Tuberculosis Infection
Source: J Clin Microbiol. 2017 Jan 25;55(2):391–402. doi: 10.1128/JCM.01646-16 (PMC5277508; doi:10.1128/JCM.01646-16)
Supplement: Supplemental material [file supp_55_2_391__index.html]

Supplemental material 

# Highly Multiplexed Proteomic Analysis of Quantiferon Supernatants To Identify Biomarkers of Latent Tuberculosis Infection

## Supplemental material

- Supplemental file 1 -

  Fig S1 (Distribution of scale factors for median normalization prior to data analysis) and S2 (EsxB detection in stimulated QFT-GIT tubes as an internal control for sample quality assessment)

  PDF, 117K
