# Supplementary material for: Highly Multiplexed Proteomic Analysis of Quantiferon Supernatants To Identify Biomarkers of Latent Tuberculosis Infection
Source: J Clin Microbiol. 2017 Jan 25;55(2):391–402. doi: 10.1128/JCM.01646-16 (PMC5277508; doi:10.1128/JCM.01646-16)
Supplement: Supplemental material [file JCM.01646-16_zjm999095333s1.pdf]

FIG S1

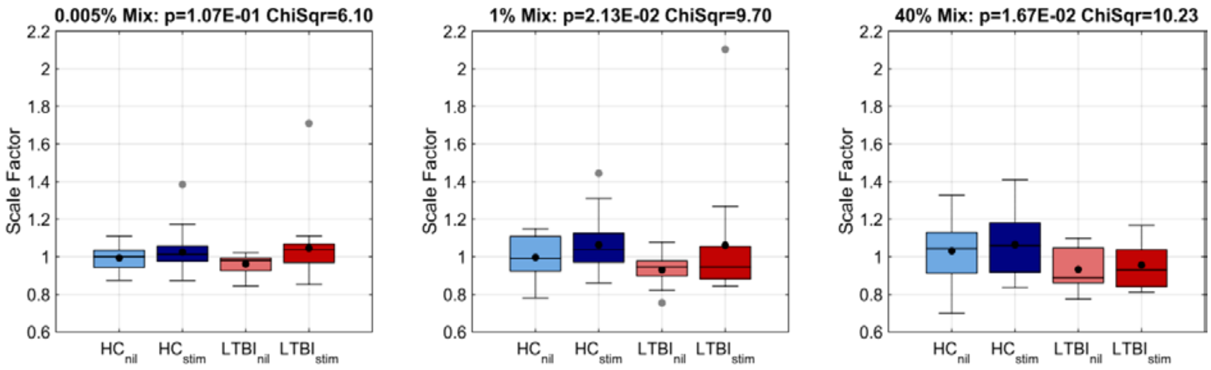

FIG S1 Distribution of scale factors for median normalization prior to data analysis. Differences in total protein abundance between the four sample groups (LTBI *vs*. HC, stimulated *vs*. nil) were not significant in any of the three sample dilutions employed in the SOMAscan assay.

FIG S2

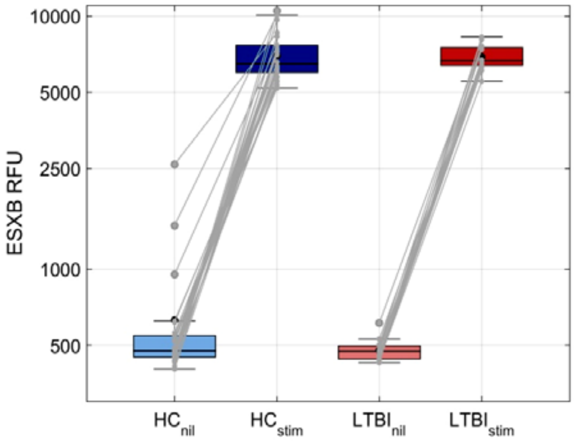

FIG S2 EsxB detection of in stimulated QFT-GIT tubes as an internal control for sample quality assessment. EsxB (ESAT-6-like protein EsxB, CFP10) is one of the Mtb antigens used to coat the QFT-GIT tubes to elicit a cell-mediated immune response compared to uncoated control (nil) tubes.

The box plots show the signals obtained with an EsxB-specific SOMAmer, and the gray lines indicate paired samples from the nil tube and stimulated tube in HC and LTBI.
